# Delineating *Microhyla ornata* (Anura, Microhylidae): mitochondrial DNA barcodes resolve century-old taxonomic misidentification

**DOI:** 10.1080/23802359.2018.1501286

**Published:** 2018-08-09

**Authors:** Sonali Garg, Abhijit Das, Rachunliu G. Kamei, S. D. Biju

**Affiliations:** a Systematics Lab, Department of Environmental Studies, University of Delhi, Delhi, India;; b Wildlife Institute of India, Dehradun, India;; c Department of Life Sciences, The Natural History Museum, London, UK

**Keywords:** 16S, cryptic species, DNA barcoding, haplotype network, species distribution

## Abstract

*Microhyla ornata*, a species originally described from the southwest coast of India in 1841, was long reported to be wide-ranging throughout South, Southeast, and East Asia. Although the name *M*. *ornata* is restricted to populations from South Asia, the species is still considered to occur widely in India and its neighboring regions. To clarify the identity and geographical distribution of *M.* ‘*ornata*’, we performed DNA barcoding using a fragment of the mitochondrial 16S rRNA gene from 62 newly obtained samples. Our results show that this taxon is restricted to Peninsular India and Sri Lanka, whereas, populations from the other parts represent three different species – *M. mukhlesuri*, *M. mymensinghensis,* and *M. nilphamariensis*, creating new country records for India. Our work reemphasizes the benefits of DNA barcoding for rapidly identifying populations of widespread species and provides insights into the patterns of genetic differentiation in the *M.* ‘*ornata*’ species complex of South Asia.

## Introduction

The Ornate narrow-mouthed frog was described as *Engystoma ornatum* Duméril and Bibron from coastal Malabar, nearly two centuries ago (Duméril and Bibron 1841). Although the type locality “côte Malabar” is located in Peninsular India, it is known to be imprecise (Biju 2001). Subsequently, *Engystoma ornatum* (=*Microhyla ornata*) was considered as a wide-ranging species occurring throughout South, Southeast, and East Asia (Matsui et al. 2005 and references therein). On the basis of populations identified as *M.* ‘*ornata*’, this common and locally abundant frog has become a widely studied microhylid species for broader biological investigations ranging from embryonic and larval development, feeding ecology, to environmental impacts and adaptation (e.g., Kumbar and Pancharatna 2001; Saidapur 2001; Kuramoto and Joshy 2006; Wells 2007; Kerby et al. 2010; Mali and Gramapurohit 2016). Despite the vast knowledge, a systematic study of populations from the entire presumed range of the species was not attempted until about a decade ago, probably due to their overall conserved morphology (e.g., Matsui et al. 2011, Hasan et al. 2012). Matsui et al. (2005) were the first to examine the genetic variations among populations representing three geographical regions—South Asia (India and Bangladesh), Southeast Asia (Thailand and Laos), and East Asia (China, including Taiwan, and Ryukyu Islands, Japan). Their study assigned the *M.* ‘*ornata*’ populations of Southeast and East Asia to two previously known species, *M. fissipes* Boulenger (Thailand, Laos, China, and Taiwan) and *M. okinavensis* Stejneger (Ryukyu Islands), and restricted the name *M.* *ornata* to the South Asian populations.

In the recent years, the taxonomy of Asian *Microhyla* frogs has undergone considerable changes with the formal description of several new species (AmphibiaWeb 2017; Frost 2017; Khatiwada et al. 2017) and insights on systematic relationships from phylogenetic studies (e.g., Matsui et al. 2011; Pyron and Wiens 2011). The South Asian *M.* ‘*ornata*’ was subsequently shown to be a species complex (Hasan et al. 2012), followed by the description of four new and closely allied species—*M. mymensinghensis* (Hasan et al. 2014), *M. mukhlesuri* (Hasan et al. 2014), *M. nilphamariensis* (Howlader et al. 2015), and *M. taraiensis* (Khatiwada et al. 2017). Although these four species are currently known to occur only in Bangladesh or/and Nepal, they have raised further confusions surrounding the taxonomic identity and geographical distribution of *M. ornata*. The latter continues to be considered as a widely distributed species throughout India and its neighboring countries like Bangladesh, Bhutan, Nepal, Pakistan, and Sri Lanka (e.g., Dutta et al. 2008; AmphibiaWeb 2017; Frost 2017), on the basis of century-old range assumptions (e.g., Boulenger 1882; Parker 1934), previous literature (e.g., Matsui et al. 2005; Matsui et al. 2011), as well as checklists and records lacking vouchers or molecular information (e.g., Dinesh et al. 2009, Mathew and Sen 2010). It has, therefore, become imperative to clarify what is *M. ornata*, identify the populations to which this name implies, and delineate the exact range of the species and its close congeners. Under such scenarios, especially with difficulties in verifying individual records or the identity of historical collections, DNA barcoding has proved to be a useful tool for species identification as well as rapid assessment of the genetic diversity (e.g., Hebert et al. 2003; Hebert and Gregory 2005; Hajibabaei et al. 2007; Crawford et al. 2013; Chambers and Hebert 2016; Estupiñán et al. 2016; Lyra et al. 2017). Using this approach, we studied DNA barcodes of the mitochondrial 16S rRNA gene generated from the newly sampled *M.* ‘*ornata*’-like populations from regions across India and compared them with the previously available molecular data, in order to resolve the long-standing confusion concerning the identity and distribution of *M. ornata*.

## Materials and methods

### Sample collection

Field surveys were conducted across the known range of *Microhyla* ‘*ornata*’ in India. A list of localities and vouchers used in the study is provided in Table S1. Sampling was mostly carried out during the breeding season, either through opportunistic visual searches or by locating calling males. After euthanization in Tricaine methanesulfonate, tissue samples were obtained from the thigh (adult and/or juvenile) or tail (tadpole) muscle, kept in absolute ethanol, and stored at −20 °C for molecular studies. Geographical coordinates were recorded with Garmin 76CSx GPS using the WGS84 datum system. Distribution maps were prepared in QGIS (http://www.qgis.org).

### DNA extraction, PCR amplification, and sequencing

Genomic DNA was extracted from 62 tissue samples using the DNeasy blood and tissue kit (Qiagen, Valencia, CA, USA) following the manufacturer’s protocol. A mitochondrial 16S rRNA gene fragment of ∼540 bp was PCR-amplified using standard protocols and previously published primers 16Sar and 16Sbr (Simon et al. 1994). This short fragment is a frequently used DNA barcode region for identification and delineation of amphibian species (e.g., Vences et al. 2005; Fouquet et al. 2007; Biju et al. 2014a, 2014b; Garg and Biju 2016, 2017; Garg et al. 2017). Cycle-sequencing was performed on both strands using BigDye Terminator v3.1 Cycle Sequencing Kit on ABI 3730 automated DNA sequencer (Applied Biosystems, Foster City, CA, USA). Sequences were assembled, checked and edited in ChromasPro v1.34 (Technelysium Pty Ltd., South Brisbane, Australia) , and deposited in the National Center for Biotechnology Information (NCBI) GenBank under accession numbers MH549575–MH549636 (Table S1).

### Molecular analyses

All the previously available 16S rRNA sequences of *Microhyla* ‘*ornata*’ and closely related congeners (*M. mukhlesuri*, *M. mymensinghensis* and *M. nilphamariensis*) were retrieved from the GenBank. Additionally, representative DNA sequences for 24 other known *Microhyla* species (one each) and an outgroup taxon (*Kaloula pulchra*) were included in the dataset. A total of 170 sequences were aligned using ClustalW in MEGA 6.0 (Tamura et al. 2013). The alignment was manually optimized and ambiguous regions were excluded for phylogenetic analyses. Maximum Likelihood (ML) and Bayesian analyses were performed using the General Time Reversible model with proportion of invariant sites and gamma-distributed rate variation among sites (GTR + I + G), which was determined as the best-fit model in Modeltest 3.4 (Posada and Crandall 1998). The ML tree was estimated using RAxML 7.3.0 (Stamatakis et al. 2008) in raxmlGUI 1.1 (Silvestro and Michalak 2012) based on a thorough ML search executed for 200 independent runs along with 1000 rapid bootstrap (BS) replicates. Bayesian analysis was performed in MrBayes (Ronquist and Huelsenbeck 2003) with four Metropolis-Coupled Markov Chain Monte Carlo (MCMCMC) runs executed for 10 million generations and sampling after every 1000 generations. Bayesian posterior probabilities (BPP) were estimated after discarding the first 2000 trees based on the burn-in value determined in Tracer v1.6 (Rambaut et al. 2014). PAUP* (Swofford 2002) was used to compute genetic distances, both uncorrected pairwise distances and those corrected using the Kimura two-parameter (K2P) model. Sequence divergences between species and among individuals of a species were calculated based on delineation of genetically identified species in the phylogenetic analyses. Further, a Median-Joining network was constructed using the software Network 4.6.1.0 (www.fluxus-engineering.com), in order to evaluate relationships and possible mutation steps among 56 haplotypes representing 147 sequences from six species—*M. fissipes*, *M. mukhlesuri*, *M. mymensinghensis*, *M. nilphamariensis*, *M. ornata,* and *M. taraiensis*.

## Results and discussion

### DNA barcoding reveals four in one species

**Figure 1.**
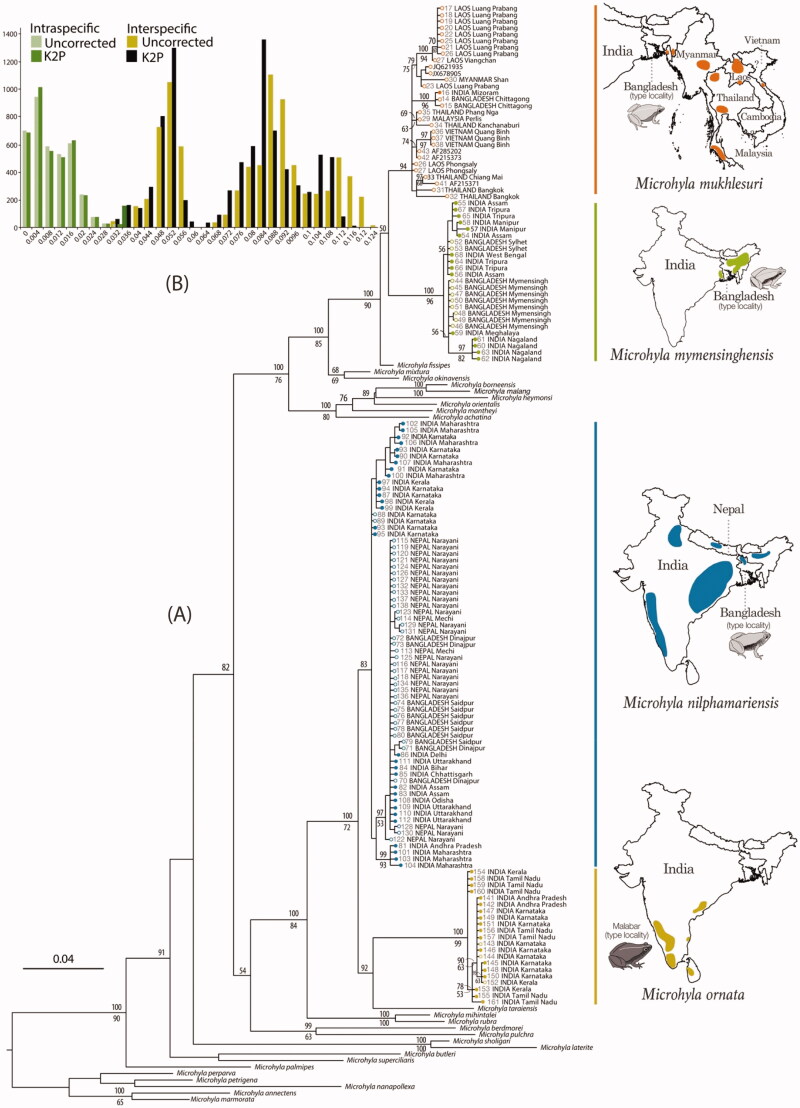
DNA barcoding based on mitochondrial 16S rRNA gene sequences (∼540 bp). (A) Maximum Likelihood RAxML tree from total 145 newly sampled and previously available populations of *Microhyla mukhlesuri*, *M. mymensinghensis*, *M. nilphamariensis,* and *M. ornata*, along with sequences representing 24 other *Microhyla* species. *Kaloula pulchra* was used as the outgroup taxon. Bayesian Posterior Probabilities and RAxML bootstrap values >50% are indicated above and below the branches, respectively. Closed circles indicate samples from the present study; open circles indicate GenBank sequences. Geographical distribution of species is shown on the right panel. (B) Frequency distribution of intra- and interspecific sequence divergences for *Microhyla mukhlesuri*, *M. mymensinghensis*, *M. nilphamariensis,* and *M. ornata*, based on uncorrected and K2P pairwise distances.

In the phylogenetic analyses, all sampled *Microhyla* ‘*ornata*’-like populations were concordantly clustered with four previously known species—*M. mukhlesuri*, *M. mymensinghensis*, *M. nilphamariensis,* and *M. ornata* (Figure 1(A)). Among these, two well-supported species groups were observed – (1) *M. mukhlesuri* and *M. mymensinghensis,* along with *M. fissipes* from Southeast and East Asia, and (2) *M. nilphamariensis* and *M. ornata,* along with *M. taraiensis* from Nepal (Figure 1(A)). At the population-level, several well-supported haplotype groups (BPP >95, BS >70) were observed, showing strong population structures within these species. Most species lineages were well-differentiated and their relationships were largely in agreement with the previous studies (e.g., Matsui et al. 2011; Hasan et al. 2012, 2014; Howlader et al. 2015; Khatiwada et al. 2017).

Our results confirm that populations previously referred to as *Microhyla* ‘*ornata*’ represent four different species. Previously, *M. ornata* was considered as a widely distributed species in South Asia, even though only a few selected populations from Karnataka (e.g., Matsui et al. 2005, 2011; Hasan et al. 2012, 2014) and a single population from northern Kerala (Howlader et al. 2015) had been genetically identified. Based on extensive sampling, we show that *M. ornata* has a narrow distribution restricted to Peninsular India (and Sri Lanka, as previously indicated by Wijayathilaka et al. 2016). More specifically in India, the presence of *M. ornata* is currently only confirmed in the states of Tamil Nadu, Kerala, Karnataka, Maharashtra, and Andhra Pradesh (Figures 1(A) and S2).

Further, our study also reveals the presence of three previously unreported *Microhyla* species in India—*M. mukhlesuri*, *M. mymensinghensis,* and *M. nilphamariensis* (Figures 1(A) and S2). *Microhyla ornata* shares a considerable part of its distribution range (in Andhra Pradesh, Maharashtra, Karnataka, and northern Kerala) with *M. nilphamariensis*; latter being the most wide-ranging member of the genus in South Asia. The presence of *M. nilphamariensis* is genetically confirmed right from the Western Ghats (Kerala, Karnataka, and Maharashtra) and Eastern Ghats (Andhra Pradesh and Odisha) up to Central India (Chhattisgarh), East India (Bihar), North India (Delhi, Uttar Pradesh, and Uttarakhand), Northeast India (Assam), Nepal, and Bangladesh. Reports of *M.* ‘*ornata*’ from Pakistan (Khan 1974) are also likely to refer to *M. nilphamariensis*. On the other hand, most of the *M.* ‘*ornata*’ populations from Northeast India belong to *M. mymensinghensis*. This species is observed to be the most common and widely distributed member across the states of Assam, Manipur, Meghalaya, Nagaland, Tripura, and West Bengal. In Assam, the range of *M. mymensinghensis* extends close to that of *M. nilphamariensis* (Figures 1(A) and S1) but the two were not observed to occur sympatrically. A third species from Bangladesh, *M. mukhlesuri*, is also confirmed to be present in the adjoining Indian regions (Mizoram). Although *M. mukhlesuri* was found at a single locality in our study, it is likely to be present in other nearby regions as evident from its wide range across Southeast Asia (Yuan et al. 2016) (Figures 1(A) and S1). Our study did not record *M. taraiensis* in India, probably due to the lack of sampling in regions adjoining Nepal.

**Figure 2.**
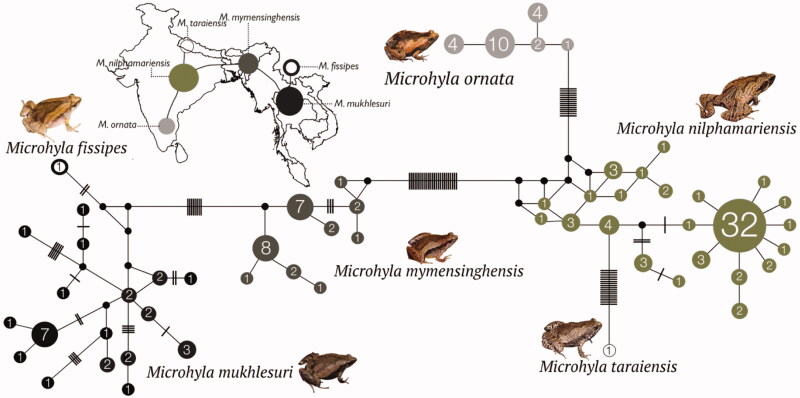
Median-Joining haplotype network based on 147 mitochondrial 16S rRNA gene sequences from six closely related *Microhyla* species. Circle sizes are proportional to the number of haplotype sequences involved, as represented with numbers inside the circles. Black circles represent median vectors. Each branch represents one mutation step; black bars represent additional mutation steps. A schematic representation of species relationships with respect to geographical distribution is shown over the map.

These findings will have implications on the conservation requirements of *Microhyla ornata*, which is currently assigned the Least Concern IUCN (International Union for Conservation of Nature) Red List status (Dutta et al. 2008) due to its presumed wide distribution in South Asia. Since this species is now found to have considerably smaller range, a reassessment of its conservation status will be necessary based on the revised distribution and subsequent verification of previous literature and records.

### Genetic differentiation within the *Microhyla* ‘*ornata*’ complex

The observed uncorrected and K2P genetic distances (Table S2) for four closely allied *Microhyla* species indicate considerable interspecific divergence among the species as well as high intraspecific divergence at the population-level. While *M. mymensinghensis*, *M. nilphamariensis,* and *M. ornata* showed intraspecific distances <2.5%, certain populations of *M. mukhlesuri* from Southeast Asia were divergent by up to 3.4%. Although genetic differentiation between species was more distinct at distances ≥3.5% with no overlap between the intra- and interspecific distances (Figure 1(B)), a wide range of interspecific distances (2.8–12.3%) was observed among these four recognized species (Table S2).

Further, the haplotype network provided insights into relationships among the haplotypes of six closely related species. The two species groups observed in our phylogenetic analyses (Figure 1(A)) were recovered as distinct clusters (Figure 2) with no haplotypes shared either among species of the same group or between members of the two groups. Genetic differentiation was relatively lower in the group comprising of *M. fissipes*, *M. mukhlesuri,* and *M. mymensinghensis*, in comparison to that consisting of *M. nilphamariensis*, *M. ornata,* and *M. taraiensis*. The two groups were connected by *M. mymensinghensis* and *M. nilphamariensis* that showed close or overlapping geographical distributions. *Microhyla ornata* (southern India) occupied a distant position with populations from Tamil Nadu (Eastern Ghats) linked to *M. nilphamariensis*; first with *M. nilphamariensis* populations from Maharashtra, Karnataka, and Kerala, followed largely by those found further north in the Indian states of Andhra Pradesh, Bihar, Delhi, Chhattisgarh, and Uttarakhand as well as Nepal and Bangladesh. However, the *M. nilphamariensis* populations from Karnataka were more closely linked to *M. taraiensis* from Nepal, whereas populations from Kerala (followed by Karnataka and Maharashtra) were close to *M. mymensinghensis* from Nagaland (followed by the remaining populations of Northeast India and Bangladesh).

Altogether, our results not only delineate *Microhyla ornata* but also clarify boundaries of closely related species in the light of their extended geographical distributions. This will facilitate future studies to decipher the patterns of gene flow and the mechanisms underlying diversification of *Microhyla* frogs in South Asia.

## Supplementary Material

Supplemental Material
