# Supplementary material for: Delineating Microhyla ornata (Anura, Microhylidae): mitochondrial DNA barcodes resolve century-old taxonomic misidentification
Source: Mitochondrial DNA B Resour. 2018 Aug 9;3(2):856–61. doi: 10.1080/23802359.2018.1501286 (PMC7800528; doi:10.1080/23802359.2018.1501286)
Supplement: Supplemental Material [file TMDN_A_1501286_SM4648.docx]

**Supplementary Information**

Mitochondrial DNA Part B, 3:2, 856-861, DOI: 10.1080/23802359.2018.1501286

Published online 09 August 2018

**Delineating *Microhyla ornata* (Anura, Microhylidae): Mitochondrial DNA barcodes resolve century old taxonomic misidentification**

Sonali Garg^a^, Abhijit Das^b^, Rachunliu G Kamei^a,c^, SD Biju^a^

^a^Systematics Lab, Department of Environmental Studies, University of Delhi, Delhi, India; ^b^Wildlife Institute of India, Chandrabani, Dehradun, Uttarakhand, India; ^c^Department of Life Sciences, The Natural History Museum, London, United Kingdom


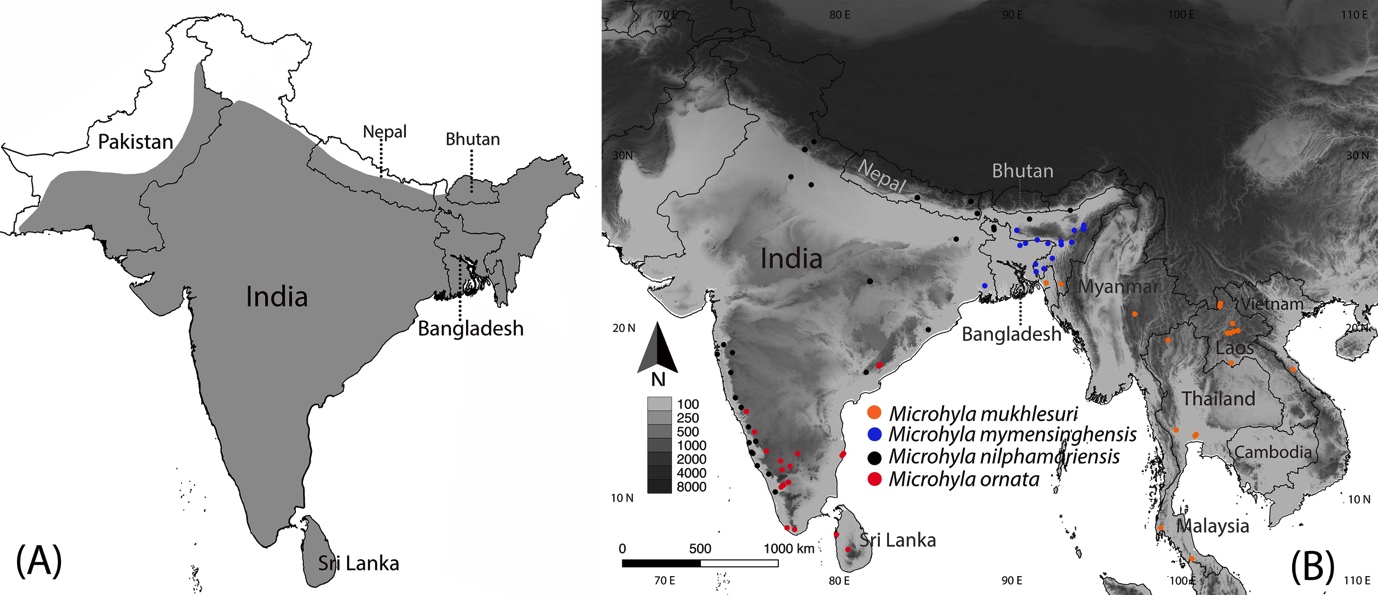


**Figure S1.** **(A)** Known distribution of *Microhyla ornata* before this study, **(B)** Revised distribution of *M. ornata* along with three morphologically and genetically related species (*M. mukhlesuri*, *M. mymensinghensis* and *M. nilphamariensis*) based on genetically confirmed samples.


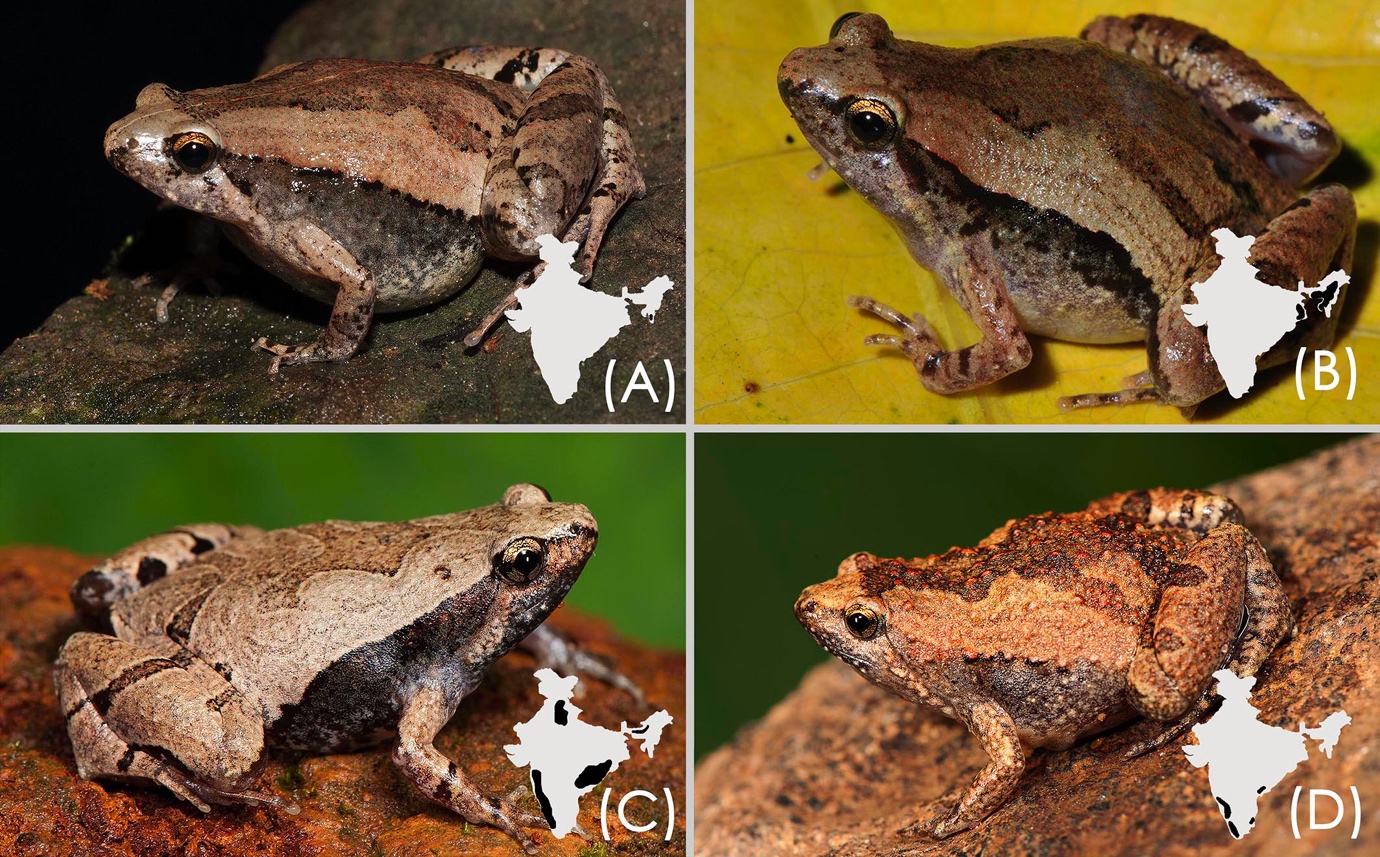


**Figure S2.** The four closely related *Microhyla* species and their distributions in India: **(A)** *M. mukhlesuri*, **(B)** *M. mymensinghensis*, **(C)** *M. nilphamariensis*, and **(D)** *M. ornata*.

**Table S1.** List of samples used in the study. Geographical coordinates are included for samples reported in the present study.

|  | Species | Locality/Coordinates | Voucher/Source | Accession No. |
| --- | --- | --- | --- | --- |
| 1 | *Microhyla achatina* | Indonesia: Java | MZB Amp 16402 | AB634656 |
| 2 | *Microhyla annectens* | Malaysia: Selangor | KUHE 53373 | AB634658 |
| 3 | *Microhyla berdmorei* | Malaysia: Selangor | KUHE 52034 | AB598338 |
| 4 | *Microhyla borneensis* | Malaysia: Sarawak | KUHE 53165 | AB598329 |
| 5 | *Microhyla butleri* | Vietnam: A Roang | KUHE 40591 | AB634664 |
| 6 | *Microhyla fissipes* | China: Anhui | KUHE 32943 | AB201185 |
| 7 | *Microhyla heymonsi* | NA | NA | AY458596 |
| 8 | *Microhyla laterite* | India: Karnataka | BNHS 5965 | KT600670 |
| 9 | *Microhyla malang* | Malaysia: Sarawak | KUHE 53018 | AB598319 |
| 10 | *Microhyla mantheyi* | Malaysia: Selangor | KUHE 15726 | AB598333 |
| 11 | *Microhyla marmorata* | Laos: Houapan | KUHE 32455 | AB634668 |
| 12 | *Microhyla mihintalei* | Sri Lanka: Anuradhapura | DZ1468 | KU214861 |
| 13 | *Microhyla mixtura* | China: Sichuan | CIB 20070248 | AB634669 |
| 14 | *Microhyla mukhlesuri* | Bangladesh: Chittagong | Morn -Bd11 (IABHU 3879) | AB543608 |
| 15 | *Microhyla mukhlesuri* | Bangladesh: Chittagong | Morn -Bd12 (IABHU 3880) | AB543609 |
| 16 | *Microhyla mukhlesuri* | India: Mizoram  (22.53°N 92.89°E) | SDBDU 2010.1332 | MH549575 |
| 17 | *Microhyla mukhlesuri* | Laos: Luang Prabang | K3361 | KR827917 |
| 18 | *Microhyla mukhlesuri* | Laos: Luang Prabang | K3090 | KR827918 |
| 19 | *Microhyla mukhlesuri* | Laos: Luang Prabang | K3087 | KR827919 |
| 20 | *Microhyla mukhlesuri* | Laos: Luang Prabang | K3334/2006.2360 | KR827920 |
| 21 | *Microhyla mukhlesuri* | Laos: Luang Prabang | K3179/2006.2362 | KR827921 |
| 22 | *Microhyla mukhlesuri* | Laos: Luang Prabang | K3216/2006.2381 | KR827922 |
| 23 | *Microhyla mukhlesuri* | Laos: Luang Prabang | K4/1997.8359 | KR827925 |
| 24 | *Microhyla mukhlesuri* | Laos: Luang Prabang | K3252/2006.2391 | KR827926 |
| 25 | *Microhyla mukhlesuri* | Laos: Luang Prabang | K3154/006.2331 | KR827931 |
| 26 | *Microhyla mukhlesuri* | Laos: Phongsaly | K1946/2005.0177 | KR827928 |
| 27 | *Microhyla mukhlesuri* | Laos: Phongsaly | K1634/2004.0426 | KR827929 |
| 28 | *Microhyla mukhlesuri* | Laos: Viangchan | 717D | KR827927 |
| 29 | *Microhyla mukhlesuri* | Malaysia: Perlis | JAM 1991 | KC822490 |
| 30 | *Microhyla mukhlesuri* | Myanmar: Shan | CAS HERP 230957 | KC179995 |
| 31 | *Microhyla mukhlesuri* | Thailand: Bangkok | 0976Y | KR827923 |
| 32 | *Microhyla mukhlesuri* | Thailand: Bangkok | KUHE 22064 | AB634666 |
| 33 | *Microhyla mukhlesuri* | Thailand: Chiang Mai | K3009 | KR827924 |
| 34 | *Microhyla mukhlesuri* | Thailand: Kanchanaburi | KUHE 35165 | AB201186 |
| 35 | *Microhyla mukhlesuri* | Thailand: Phang Nga | P306 | KR827930 |
| 36 | *Microhyla mukhlesuri* | Vietnam: Quang Binh | ZFMK86426 | EU157074 |
| 37 | *Microhyla mukhlesuri* | Vietnam: Quang Binh | ZFMK86370 | EU157075 |
| 38 | *Microhyla mukhlesuri* | Vietnam: Quang Binh | ZFMK86752 | EU157076 |
| 39 | *Microhyla mukhlesuri* | NA | KIZHERP0138 | JX678905 |
| 40 | *Microhyla mukhlesuri* | NA | NA | JQ621935 |
| 41 | *Microhyla mukhlesuri* | NA | NA | AF215371 |
| 42 | *Microhyla mukhlesuri* | NA | NA | AF215373 |
| 43 | *Microhyla mukhlesuri* | NA | TZ52 | AF285202 |
| 44 | *Microhyla mymensinghensis* | Bangladesh: Mymensingh | Morn -Bd1 | AB530529 |
| 45 | *Microhyla mymensinghensis* | Bangladesh: Mymensingh | Morn -Bd2 | AB530530 |
| 46 | *Microhyla mymensinghensis* | Bangladesh: Mymensingh | Morn -Bd3 | AB530531 |
| 47 | *Microhyla mymensinghensis* | Bangladesh: Mymensingh | Morn -Bd4 | AB530532 |
| 48 | *Microhyla mymensinghensis* | Bangladesh: Mymensingh | Morn -Bd5 | AB530533 |
| 49 | *Microhyla mymensinghensis* | Bangladesh: Mymensingh | Morn -Bd6 | AB530534 |
| 50 | *Microhyla mymensinghensis* | Bangladesh: Mymensingh | Morn -Bd7 | AB530535 |
| 51 | *Microhyla mymensinghensis* | Bangladesh: Mymensingh | Morn -Bd8 (DFBGBAU 306) | AB530536 |
| 52 | *Microhyla mymensinghensis* | Bangladesh: Sylhet | Morn -Bd9 (IABHU 3898) | AB543606 |
| 53 | *Microhyla mymensinghensis* | Bangladesh: Sylhet | Morn -Bd10 (IABHU 3899) | AB543607 |
| 54 | *Microhyla mymensinghensis* | India: Assam  (24.79°N 92.79°E) | SDBDU 2008.1321 | MH549576 |
| 55 | *Microhyla mymensinghensis* | India: Assam  (24.98°N 92.78°E) | ADWII_BM1 | MH549577 |
| 56 | *Microhyla mymensinghensis* | India: Assam  (24.98°N 92.78°E) | ADWII_BM2 | MH549578 |
| 57 | *Microhyla mymensinghensis* | India: Manipur  (24.93°N 93.39°E) | SDBDU 2007.14 | MH549579 |
| 58 | *Microhyla mymensinghensis* | India: Manipur  (24.94°N 93.40°E) | SDBDU 2008.1440 | MH549580 |
| 59 | *Microhyla mymensinghensis* | India: Meghalaya  (25.62°N 90.23°E) | SDBDU 2008.1360 | MH549581 |
| 60 | *Microhyla mymensinghensis* | India: Nagaland  (25.71°N 94.05°E) | SDBDU 2007.30 | MH549582 |
| 61 | *Microhyla mymensinghensis* | India: Nagaland  (25.68°N 94.12°E) | SDBDU 2007.209 | MH549583 |
| 62 | *Microhyla mymensinghensis* | India: Nagaland  (25.92°N 94.09°E) | SDBDU 2007.248 | MH549584 |
| 63 | *Microhyla mymensinghensis* | India: Nagaland  (25.62°N 93.54°E) | SDBDU 2009.37 | MH549585 |
| 64 | *Microhyla mymensinghensis* | India: Tripura  (23.41°N 91.79°E) | SDBDU 2009.568 | MH549586 |
| 65 | *Microhyla mymensinghensis* | India: Tripura  (24.00°N 92.28°E) | SDBDU 2009.611 | MH549587 |
| 66 | *Microhyla mymensinghensis* | India: Tripura  (23.23°N 91.34°E) | SDBDU 2009.482 | MH549588 |
| 67 | *Microhyla mymensinghensis* | India: Tripura  (23.66°N 91.31°E) | SDBDU 2009.444 | MH549589 |
| 68 | *Microhyla mymensinghensis* | India: West Bengal  (22.43°N 88.38°E) | SDBDU 2015.2904 | MH549590 |
| 69 | *Microhyla nanapollexa* | Vietnam: Quang Nam | PT-484 | KM509164 |
| 70 | *Microhyla nilphamariensis* | Bangladesh: Dinajpur | DB-Hi-FROG 12005 | AB201187 |
| 71 | *Microhyla nilphamariensis* | Bangladesh: Dinajpur | Morn -Bd1 (IABHU 22135) | AB530537 |
| 72 | *Microhyla nilphamariensis* | Bangladesh: Dinajpur | Morn -Bd2 (IABHU 22136) | AB530538 |
| 73 | *Microhyla nilphamariensis* | Bangladesh: Dinajpur | Morn -Bd3 (IABHU 22137) | AB530539 |
| 74 | *Microhyla nilphamariensis* | Bangladesh: Saidpur | MZH-2360 | KP072787 |
| 75 | *Microhyla nilphamariensis* | Bangladesh: Saidpur | MZH-2361 | KP072788 |
| 76 | *Microhyla nilphamariensis* | Bangladesh: Saidpur | MZH-2362 | KP072789 |
| 77 | *Microhyla nilphamariensis* | Bangladesh: Saidpur | MZH-2363 | KP072790 |
| 78 | *Microhyla nilphamariensis* | Bangladesh: Saidpur | MZH-2364 | KP072791 |
| 79 | *Microhyla nilphamariensis* | Bangladesh: Saidpur | MZH-2365 | KP072792 |
| 80 | *Microhyla nilphamariensis* | Bangladesh: Saidpur | MZH-2366 | KP072793 |
| 81 | *Microhyla nilphamariensis* | India: Andhra Pradesh  (81.54°N 17.43°E) | SDBDU 2007.4987 | MH549591 |
| 82 | *Microhyla nilphamariensis* | India: Assam  (26.27°N 90.96°E) | SDBDU 2015.2905 | MH549592 |
| 83 | *Microhyla nilphamariensis* | India: Assam  (26.77°N 93.31°E) | SDBDU 2015.2915 | MH549593 |
| 84 | *Microhyla nilphamariensis* | India: Bihar  (25.11°N 86.73°E) | SDBDU 2011.869 | MH549594 |
| 85 | *Microhyla nilphamariensis* | India: Chhattisgarh  (22.71°N 81.76°E) | SDBDU 2010.401 | MH549595 |
| 86 | *Microhyla nilphamariensis* | India: Delhi  (28.71°N 77.19°E) | SDBDU 2016.3375A | MH549596 |
| 87 | *Microhyla nilphamariensis* | India: Karnataka | NA | AB530627 |
| 88 | *Microhyla nilphamariensis* | India: Karnataka | BNHS 5028 | AB530628 |
| 89 | *Microhyla nilphamariensis* | India: Karnataka | BNHS 5029 | AB530629 |
| 90 | *Microhyla nilphamariensis* | India: Karnataka | RBRL 040723-04 | AB530630 |
| 91 | *Microhyla nilphamariensis* | India: Karnataka  (15.40°N 74.33°E) | SDBDU 2011.836 | MH549597 |
| 92 | *Microhyla nilphamariensis* | India: Karnataka  (14.27°N 74.75°E) | SDBDU 2011.1376 | MH549598 |
| 93 | *Microhyla nilphamariensis* | India: Karnataka  (12.82°N 74.93°E) | SDBDU 2015.3045 | MH549599 |
| 94 | *Microhyla nilphamariensis* | India: Karnataka  (13.36°N 74.79°E) | SDBDU 2015.3060 | MH549600 |
| 95 | *Microhyla nilphamariensis* | India: Karnataka  (12.75°N 75.01°E) | SDBDU 2017.3600 | MH549601 |
| 96 | *Microhyla nilphamariensis* | India: Karnataka  (75.17°N 13.44°E) | SDBDU 2003.1353 | MH549602 |
| 97 | *Microhyla nilphamariensis* | India: Kerala  (11.55°N 75.92°E) | SDBDU 2008.403 | MH549603 |
| 98 | *Microhyla nilphamariensis* | India: Kerala  (10.52°N 76.29°E) | SDBDU 2003.1345 | MH549604 |
| 99 | *Microhyla nilphamariensis* | India: Kerala  (12.04°N 75.26°E) | SDBDU 2017.3581 | MH549605 |
| 100 | *Microhyla nilphamariensis* | India: Maharashtra  (17.42°N 73.73°E) | SDBDU 2004.4507 | MH549606 |
| 101 | *Microhyla nilphamariensis* | India: Maharashtra  (18.46°N 72.95°E) | SDBDU 2011.1459 | MH549607 |
| 102 | *Microhyla nilphamariensis* | India: Maharashtra  (15.96°N 73.99°E) | SDBDU 2014.2482 | MH549608 |
| 103 | *Microhyla nilphamariensis* | India: Maharashtra  (18.56°N 73.83°E) | SDBDU 2014.2676 | MH549609 |
| 104 | *Microhyla nilphamariensis* | India: Maharashtra  (19.02°N 73.32°E) | SDBDU 2014.2750 | MH549610 |
| 105 | *Microhyla nilphamariensis* | India: Maharashtra  (15.96°N 73.99°E) | SDBDU 2002.1336 | MH549611 |
| 106 | *Microhyla nilphamariensis* | India: Maharashtra  (15.96°N 73.99°E) | SDBDU 2004.1433 | MH549612 |
| 107 | *Microhyla nilphamariensis* | India: Maharashtra  (17.42°N 73.73°E) | SDBDU 2007.1562 | MH549613 |
| 108 | *Microhyla nilphamariensis* | India: Odisha  (19.89°N 85.13°E) | SDBDU 2015.3121 | MH549614 |
| 109 | *Microhyla nilphamariensis* | India: Uttarakhand  (30.28°N 77.97°E) | ADWII_DW1 | MH549615 |
| 110 | *Microhyla nilphamariensis* | India: Uttarakhand  (30.28°N 78.01°E) | ADWII_DT1 | MH549616 |
| 111 | *Microhyla nilphamariensis* | India: Uttarakhand  (30.73°N 78.53°E) | ADWII_ACC059 | MH549617 |
| 112 | *Microhyla nilphamariensis* | India: Uttar Pradesh  (28.25°N 78.37°E) | ADWII_M03 (RJM3F) | MH549618 |
| 113 | *Microhyla nilphamariensis* | Nepal: Mechi | JRK201528 | KY655950 |
| 114 | *Microhyla nilphamariensis* | Nepal: Mechi | JRK201529 | KY655951 |
| 115 | *Microhyla nilphamariensis* | Nepal: Narayani | JRK201501 | KY655926 |
| 116 | *Microhyla nilphamariensis* | Nepal: Narayani | JRK201502 | KY655927 |
| 117 | *Microhyla nilphamariensis* | Nepal: Narayani | JRK201503 | KY655928 |
| 118 | *Microhyla nilphamariensis* | Nepal: Narayani | JRK201504 | KY655929 |
| 119 | *Microhyla nilphamariensis* | Nepal: Narayani | JRK201505 | KY655930 |
| 120 | *Microhyla nilphamariensis* | Nepal: Narayani | JRK201506 | KY655931 |
| 121 | *Microhyla nilphamariensis* | Nepal: Narayani | JRK201507 | KY655932 |
| 122 | *Microhyla nilphamariensis* | Nepal: Narayani | JRK201508 | KY655933 |
| 123 | *Microhyla nilphamariensis* | Nepal: Narayani | JRK201509 | KY655934 |
| 124 | *Microhyla nilphamariensis* | Nepal: Narayani | JRK201510 | KY655935 |
| 125 | *Microhyla nilphamariensis* | Nepal: Narayani | JRK201511 | KY655936 |
| 126 | *Microhyla nilphamariensis* | Nepal: Narayani | JRK201512 | KY655937 |
| 127 | *Microhyla nilphamariensis* | Nepal: Narayani | JRK201513 | KY655938 |
| 128 | *Microhyla nilphamariensis* | Nepal: Narayani | JRK201514 | KY655939 |
| 129 | *Microhyla nilphamariensis* | Nepal: Narayani | JRK201515 | KY655940 |
| 130 | *Microhyla nilphamariensis* | Nepal: Narayani | JRK201516 | KY655941 |
| 131 | *Microhyla nilphamariensis* | Nepal: Narayani | JRK201517 | KY655942 |
| 132 | *Microhyla nilphamariensis* | Nepal: Narayani | JRK201518 | KY655943 |
| 133 | *Microhyla nilphamariensis* | Nepal: Narayani | JRK201519 | KY655944 |
| 134 | *Microhyla nilphamariensis* | Nepal: Narayani | JRK201520 | KY655945 |
| 135 | *Microhyla nilphamariensis* | Nepal: Narayani | JRK201521 | KY655946 |
| 136 | *Microhyla nilphamariensis* | Nepal: Narayani | JRK201522 | KY655947 |
| 137 | *Microhyla nilphamariensis* | Nepal: Narayani | JRK201523 | KY655948 |
| 138 | *Microhyla nilphamariensis* | Nepal: Narayani | JRK201524 | KY655949 |
| 139 | *Microhyla okinavensis* | Japan: Okinawa | IABHU5263 | AB303950 |
| 140 | *Microhyla orientalis* | Indonesia: Bali | KUHE 55073 | AB781469 |
| 141 | *Microhyla ornata* | India: Andhra Pradesh  (17.66°N 82.22°E) | SDBDU 2015.2898 | MH549619 |
| 142 | *Microhyla ornata* | India: Andhra Pradesh  (17.68°N 82.58°E) | SDBDU 2015.2899 | MH549620 |
| 143 | *Microhyla ornata* | India: Karnataka | ZSIK-A9119 | AB201188 |
| 144 | *Microhyla ornata* | India: Karnataka | BNHS 5036 | AB530632 |
| 145 | *Microhyla ornata* | India: Karnataka  (11.80°N 76.69°E) | SDBDU 2007.6029 | MH549621 |
| 146 | *Microhyla ornata* | India: Karnataka  (13.98°N 75.11°E) | SDBDU 2003.1352 | MH549622 |
| 147 | *Microhyla ornata* | India: Karnataka  (15.17°N 74.63°E) | SDBDU 2011.1314 | MH549623 |
| 148 | *Microhyla ornata* | India: Karnataka  (12.32°N 76.60°E) | SDBDU 2012.1960 | MH549624 |
| 149 | *Microhyla ornata* | India: Karnataka  (12.89°N 75.78°E) | SDBDU 2012.2198 | MH549625 |
| 150 | *Microhyla ornata* | India: Karnataka  (12.00°N 77.14°E) | SDBDU 2014.2539 | MH549626 |
| 151 | *Microhyla ornata* | India: Karnataka  (12.73°N 77.58°E) | SDBDU 2014.2555 | MH549627 |
| 152 | *Microhyla ornata* | India: Kerala | RGCB15059 | KP072794 |
| 153 | *Microhyla ornata* | India: Kerala  (8.45°N 76.98°E) | SDBDU 2015.2970 | MH549628 |
| 154 | *Microhyla ornata* | India: Kerala  (10.80°N 76.64°E) | SDBDU 2003.1344 | MH549629 |
| 155 | *Microhyla ornata* | India: Tamil Nadu  (8.36°N 77.41°E) | SDBDU 2008.1958 | MH549630 |
| 156 | *Microhyla ornata* | India: Tamil Nadu  (12.63°N 80.15°E) | SDBDU 2014.2733A | MH549631 |
| 157 | *Microhyla ornata* | India: Tamil Nadu  (12.63°N 80.15°E) | SDBDU 2014.2733B | MH549632 |
| 158 | *Microhyla ornata* | India: Tamil Nadu  (11.08°N 77.06°E) | SDBDU 2014.2794 | MH549633 |
| 159 | *Microhyla ornata* | India: Tamil Nadu  (10.92°N 76.78°E) | SDBDU 2014.2820 | MH549634 |
| 160 | *Microhyla ornata* | India: Tamil Nadu  (11.08°N 77.06°E) | SDBDU 2003.1341 | MH549635 |
| 161 | *Microhyla ornata* | India: Tamil Nadu  (11.08°N 77.06°E) | SDBUDU 2008.1720 | MH549636 |
| 162 | *Microhyla palmipes* | Indonesia: Bali | MZB Amp 16255 | AB634670 |
| 163 | *Microhyla perparva* | Malaysia: Sarawak | KUHE 53675 | AB634673 |
| 164 | *Microhyla petrigena* | Malaysia: Sarawak | KUHE 53743 | AB634675 |
| 165 | *Microhyla pulchra* | China: Guangdong | NA | KF798195 |
| 166 | *Microhyla rubra* | India: Karnataka | NA | AB201192 |
| 167 | *Microhyla sholigari* | India | VUB 0066 | AF249060 |
| 168 | *Microhyla superciliaris* | Malaysia: Negeri Sembilan | KUHE 53371 | AB634683 |
| 169 | *Microhyla taraiensis* | Nepal: Mechi | JRK201525 | KY655952 |
|  | **Outgroup** |  |  |  |
| 170 | *Kaloula pulchra* | Thailand: Kanchanaburi | KUHE 35171 | AB201194 |

**Table S2.** Inter and intraspecific uncorrected p-distances (in percent) for the mitochondrial 16S rRNA gene sequences. N1 represents the number of samples per species; N2 represents number of pairwise comparisons between species.

| Species |  |  | Uncorrected P-distances | | | K2P distances | | |
| --- | --- | --- | --- | --- | --- | --- | --- | --- |
| **Intraspecific** |  | N1 | Mean | Min | Max | Mean | Min | Max |
| *Microhyla mukhlesuri* |  | 30 | 1.5 | 0 | 3.3 | 1.5 | 0 | 3.4 |
| *Microhyla mymensinghensis* |  | 25 | 0.7 | 0 | 2.1 | 0.7 | 0 | 2.2 |
| *Microhyla nilphamariensis* |  | 69 | 0.7 | 0 | 2.3 | 0.7 | 0 | 2.4 |
| *Microhyla ornata* |  | 21 | 0.2 | 0 | 0.8 | 0.2 | 0 | 0.8 |
| **Interspecific** |  | N2 | Mean | Min | Max | Mean | Min | Max |
| *Microhyla mukhlesuri* | *Microhyla mymensinghensis* | 750 | 4.1 | 2.8 | 5.4 | 4.2 | 2.8 | 5.6 |
| *Microhyla mukhlesuri* | *Microhyla nilphamariensis* | 2070 | 9.2 | 7.1 | 10.8 | 9.9 | 7.5 | 11.7 |
| *Microhyla mukhlesuri* | *Microhyla ornata* | 630 | 9.8 | 8.1 | 11.3 | 10.5 | 8.6 | 12.3 |
| *Microhyla mymensinghensis* | *Microhyla nilphamariensis* | 1725 | 7.7 | 6.2 | 8.6 | 8.1 | 6.4 | 9.1 |
| *Microhyla mymensinghensis* | *Microhyla ornata* | 525 | 8.4 | 7.7 | 9.2 | 8.9 | 8.1 | 9.8 |
| *Microhyla nilphamariensis* | *Microhyla ornata* | 1449 | 4.8 | 3.9 | 5.7 | 5.0 | 4.0 | 5.9 |
